# Correction: Giri et al. Improving Protein–Ligand Interaction Modeling with cryo-EM Data, Templates, and Deep Learning in 2021 Ligand Model Challenge. *Biomolecules* 2023, *13*, 132

**DOI:** 10.3390/biom14111404

**Published:** 2024-11-04

**Authors:** Nabin Giri, Jianlin Cheng

**Affiliations:** Department of Electrical Engineering and Computer Science, University of Missouri, Columbia, MO 65211, USA; ngzvh@missouri.edu

There was an error in the original publication [1]. There are two references [8,9] in the original version, which do not genuinely enhance our paper.

A correction has been made to Introduction, Paragraph Number: 3.

The corrected sentence in the paragraph is as follows: One of the most popular approaches to modeling the protein–ligand complexes is molecular docking [4–7], which uses physics- or statistical potential-based molecular simulations to generate protein–ligand complex models and a scoring function for the estimation of their binding affinities to rank them.

With this correction, the order of some references has been adjusted accordingly. The authors state that the scientific conclusions are unaffected. This correction was approved by the academic editor. The original publication has also been updated.
